# Cross-Plant Few-Shot Attack Recognition and Detection in Industrial Control Systems

**DOI:** 10.3390/s26206360

**Published:** 2026-10-09

**Authors:** Mohammed Almansour, Iftikhar Ahmad, Madini O. Alassafi

**Affiliations:** Department of Information Technology, Faculty of Computing and Information Technology, King Abdulaziz University, Jeddah 21589, Saudi Arabia; iakhan@kau.edu.sa (I.A.); malasafi@kau.edu.sa (M.O.A.)

**Keywords:** industrial control systems, intrusion detection, few-shot learning, cross-plant transfer, anomaly scoring, baseline validity, SWaT, WADI

## Abstract

Intrusion detectors for industrial control systems are trained on the few testbeds that provide labelled attacks and then applied to plants with different sensors and processes. This study evaluates bidirectional few-shot transfer between the SWaT and WADI water testbeds, with attack identities separated across episodic splits, chronologically separated detection streams, and no gradient updates on the target plant. Cross-plant recognition reached macro-F1 scores of 0.8625 for SWaT to WADI and 0.8528 for WADI to SWaT from five labelled windows per class, but a randomly initialised encoder of the same architecture did not differ detectably on identical episodes, so these values reflect the episodic task more than the transferred representation. Detection depended on the scoring rule and on the target plant. With SWaT as the target, replacing centroid scoring with a local-neighbourhood rule raised ROC-AUC from 0.402 to 0.742 and from 0.528 to 0.797 with an untrained encoder; as the normal reference grew from one to 500 windows, the local rule rose from 0.495 to 0.805 while the centroid fell to 0.397, consistent with the mean of multimodal normal operation lying between operating states. From SWaT to WADI, neither rule exceeded chance. Cross-plant results on these benchmarks should be reported against reference conditions that carry no transferred information.

## 1. Introduction

Labelled attacks on industrial control systems are scarce for a structural reason: staging one on a running water-treatment plant or power substation is expensive, disruptive, and in most jurisdictions prohibited. The field’s response has been to train where labelled attacks exist and deploy where they do not. Whether that works is almost always posed as a binary question, whether the detector still fires on the new plant, and the answer is almost as consistently that it does not fire well.

The binary framing omits the question an operator actually faces. A detector that fires produces an alarm; a detector that identifies which attack it is seeing produces the beginning of a response. That distinction separates a transferred model that supports a control room from one that adds to its alert burden. At the level of individual attack identities, cross-plant performance appears not to have been measured.

We measure it in both directions between two heterogeneous water testbeds, SWaT (51 process channels) and WADI (123), evaluating attack-type recognition from five labelled windows per class with no target-domain gradient updates, alongside binary detection on the same encoders, under a class-disjoint attack partition and a chronologically split normal pool. Partition integrity is verified programmatically rather than asserted, and the verification output is available from the authors on request.

Recognition performance is high, but it cannot be attributed to the transfer. A source-trained encoder reaches 0.8625 macro-F1 on WADI and 0.8528 on SWaT from five labelled windows per class, against 0.8908 and 0.8310 within-plant. Measured against an untrained encoder of the same architecture on identical episodes, however, the source-trained encoder does not differ detectably in either cross-plant direction, and removing the correspondence between channel positions leaves most of the performance intact. The figures characterise the episodic protocol on these corpora rather than the quality of the transferred representation.

Detection does not transfer under the rule most prototype-based detectors inherit. Scoring a window by its distance to the mean of a target-plant normal reference performs no better than chance in either direction, and when SWaT is the target, it deteriorates as the reference grows, from 0.495 with a single reference window to 0.397 with five hundred. A method whose performance falls as data accumulates is not merely underpowered; it is estimating a quantity that does not carry the information being sought.

The explanation lies in the scoring rule rather than the learned representation. When SWaT is the target, a local-density estimate over identical embeddings reverses the trend, rising from 0.495 to 0.805 over the same range and improving in ten seeds out of ten; in the headline configuration it moves ROC-AUC from 0.402 to 0.742, and an untrained encoder shows a comparable separation. Normal operation in a process plant is multimodal, since the plant cycles through discrete operating states, and the mean of a multimodal distribution can fall between modes, in a region corresponding to no state the plant occupies. The more data used to estimate that mean, the more precisely it converges on a location of no diagnostic value. Adding centres, the usual remedy for an inadequate single centre, does not recover performance (0.519); scoring against the local neighbourhood of the reference does. From SWaT to WADI, no rule we tested rises above chance.

The scoring result is therefore tied to the target plant, but the mechanism behind it is not. Distance to a prototype is the default in few-shot classification and prototype-based anomaly detection alike, adopted by inheritance rather than examined, and our findings indicate that it can be actively harmful when the reference class is multimodal. For industrial process data, that is a common case, and whether it holds for a given plant can be checked from normal data alone before a scoring rule is chosen.

The contributions of this paper are as follows.

1. A bidirectional, attack-identifier-level few-shot transfer protocol for ICS testbeds is released with a 22-assertion programmatic partition audit and a cross-process determinism gate. Existing work on this plant pair transfers binary anomaly detectors and retrains on a portion of the target’s training data [1]; existing few-shot prototype work at attack-type granularity evaluates SWaT, WADI and a gas pipeline corpus as independent benchmarks rather than as source and target [2].
2. Cross-plant attack-type recognition is quantified at 0.8625 and 0.8528 macro-F1 from five labelled windows per class over 39 seeds under enforced determinism and placed against reference conditions carrying no transferred information, which reproduce it.
3. There is evidence that cross-plant binary detection is governed by the scoring rule rather than by the representation: when SWaT is the target, a local-neighbourhood rule exceeds centroid scoring by 0.340 ROC-AUC over identical embeddings, a separation that persists at 0.269 when the embeddings come from an untrained network, while centroid performance degrades as the target-plant normal reference grows; from SWaT to WADI, neither rule exceeds chance.
4. We characterise what each capability requires from the target plant and demonstrate that cross-plant results on these benchmarks should be reported alongside representation-free reference conditions that establish what they measure.

We additionally report a pre-registered self-supervised pretraining ablation that improved all four recognition settings without reaching significance in any (Section 4.4), included because it was pre-registered and reporting only the successful portion of a declared confirmatory family would defeat the purpose of declaring one.

## 2. Related Work

Two research programmes bear on the question this paper asks, and they have developed largely in parallel. One studies how detectors learn from a handful of labelled attacks, almost always within a single corpus. The other studies what happens when a detector is moved between datasets, almost always with the task reduced to a binary decision. Adaptation and relocation, in other words, have been investigated separately, and the combination has received little attention. A third thread, smaller and more technical, concerns how anomaly scores are computed once a representation exists; that thread turns out to matter more than its visibility in the literature would suggest, and we treat it last.

### 2.1. Adaptation Within a Corpus

Few-shot intrusion detection began as a response to class imbalance and has broadened into a general treatment of label scarcity. Siamese formulations recast detection as similarity learning, which suits a regime in which only a few examples of any new attack exist: Wang et al. pair capsule representations with subtype sampling under imbalance and unknown-attack conditions [3], Hindy et al. demonstrate one-shot detection that reduces dependence on large labelled datasets [4], and Nafis et al. extend pair construction to zero-day threats on CICIDS2017 [5]. Pair-based methods scale awkwardly, however, since the number of pairs grows with the square of the attack vocabulary, and pairwise similarity says nothing in itself about how class semantics should survive a change in taxonomy.

Prototypical networks address the second objection by making class representation explicit. Sun et al. combine prototypical classification with capsule features, attention, and distance voting [6]; PTN-IDS applies the architecture directly where malicious samples are limited [7]; and Ahmad et al. introduce stochastic prototypes for in-vehicle traffic, adding uncertainty awareness [8]. Meta-learning pursues the same goal through the optimiser rather than the metric, with MAML applied to IoT intrusion detection [9], MASiNet developing a framework for the same domain [10], MACML integrating attention and convolution with optimisation-based meta-learning [11], and Shi et al. adding cross-attention for unseen traffic types [12]. Further variants substitute the supervision signal itself: label-free self-supervision for new and few-shot attacks [13], generative augmentation of scarce attack samples [14], federated graph contrastive learning under privacy constraints [15], and multimodal fusion of traffic graphs with network features [16]. A related literature formalises the incremental case, learning new attack classes without full retraining [17,18], which shares our premise that closed-set evaluation is unrealistic while addressing class arrival within a single stream rather than movement between plants.

What unites this work is an evaluation convention rather than a method. Novelty is nearly always simulated by holding out classes from the same corpus, so support and query sets share a feature-generation pipeline, a labelling ontology, and a collection environment. That design measures class novelty. It does not measure distribution shift, and the two are routinely conflated when results are described as addressing zero-day threats.

### 2.2. Relocation Between Datasets

The complementary literature takes shift seriously and reports discouraging results. Cantone et al. examine generalisation across network intrusion detection datasets and argue that cross-network performance is a precondition for deployment rather than an optional robustness check [19]. Elangovan et al. evaluate temporal and cross-domain robustness across public datasets spanning 2017 to 2024 and observe degradation under both [20]. De Oliveira et al. approach cross-dataset capability as a systems problem [21], and Noor et al. survey transfer learning for intrusion detection in 5G and beyond [22].

The finding is consistent: detectors that perform well within a corpus perform poorly outside it. The framing, however, is uniformly binary. These studies ask whether the alarm still fires after relocation, never whether the model still knows what it is looking at. A detector could in principle lose its calibration for normality while retaining its ability to discriminate among attack types, and nothing in this literature would detect that, because nothing in it measures attack identity after transfer.

### 2.3. The SWaT and WADI Pair

Process-level anomaly detection on these two testbeds has its own substantial literature, dominated by reconstruction and forecasting. Composite autoencoders predict and reconstruct simultaneously [23]; comparative studies place autoencoders and LSTMs above tree-based and rule-based detectors [24]; isolation-forest ensembles train semi-supervised on normal operation alone [25]. More recent work adds structural priors through spatiotemporal graph attention with periodicity extraction [26], attention-driven recurrent architectures with feature reduction [27], and digital-twin residuals fused with deep ensembles [28], while a 1D-CNN spanning SWaT, WADI, and MATPOWER reports detection of false-data-injection attacks under a shared pipeline [29].

Here, too, the task is usually binary. The attack identities that iTrust documents are typically used to construct a label sequence and then discarded, though exceptions exist: CPAC-Net classifies SWaT readings into seven attack classes [30], and factorisation-machine attribution ranks root causes across both datasets [31]. Both operate within a single plant.

Two studies sit closer to our question. MENDEL maps reduced feature spaces between ICS datasets by PCA and retrains a pre-trained detector on part of the target’s training data, reporting F1 of 0.72 for SWaT to WADI with 10% of WADI’s training set against 0.44 without adaptation [1]; it detects rather than identifies, and it updates on target data rather than forming prototypes from five labelled windows. DPL-FSAD learns decoupled prototypes with orthogonality constraints separating attack types and improves over Siamese baselines on SWaT, WADI and a gas pipeline corpus [2], evaluating the three datasets as independent benchmarks. What remains unexamined is the arrangement we adopt: one plant as source, the other as target, in both directions, with the encoder never updated on the target.

Evaluation practice on these benchmarks is itself under revision. Heydari et al. show that detector rankings on SWaT and WADI invert once an alarm budget is imposed and that methods reporting near-zero miss rates can impose more than ten times the intended alert workload [32]. Their conclusion, that threshold-free metrics quoted without operator constraints may be unusable in practice, arrives from a different direction at something close to our own result concerning source-calibrated thresholds (Section 5.4).

### 2.4. The Provenance of the Centroid Rule

Our central finding concerns none of the above architectures. It concerns a scoring convention that all of them inherit, so the convention is worth tracing.

Deep SVDD gives the modern form: a network maps normal data inside a minimum-volume hypersphere, and the anomaly score is the distance from a single learned centre [33]. The formulation has been extended in several directions, including a joint autoencoder reconstruction term to prevent hypersphere collapse [34], maximum-margin objectives with learnable centres for interpretability [35], and outlier-exposure sets that shape the decision boundary [36]. The few-shot literature reaches the same place independently, since a prototypical network’s class mean is a single centre by another name (Section 2.1). Both lineages, arriving from different problems, score by distance to one point.

That a single centre is inadequate for a structured normal class is known, and the field has a standard answer: add more centres. THOC builds multiple hyperspheres by hierarchical clustering under a multiscale objective, capturing temporal dynamics at several resolutions [37]. Multi-class Deep SVDD assigns a hypersphere per inlier category [38], and multi-centre variants pursue the same idea under a reconstruction constraint [39]. A graph-regularised formulation couples a DeepSAD hypersphere with a latent nearest-neighbour graph, though test-time scoring still reduces to one distance computation [40].

The diagnosis, then, is established. The prescription is what we find wanting. Our nearest-mode scorer is exactly this remedy, with centres fitted by *k*-means and their number chosen by silhouette, and in the WADI-to-SWaT direction it does not recover performance (0.519, Section 4.3), whereas a local-neighbourhood estimate does (0.742). Adding centres is insufficient. What succeeds is scoring a window against its nearest reference windows instead of against any set of fitted mean locations.

Our pretraining arm belongs to a further strand. It follows SimCLR [41], which showed that contrastive learning can match supervised representation quality and that augmentation composition is the decisive design choice. Adaptations to time series include DTW-based warping [42], weak and strong temporal augmentations with contextual contrasting [43], mixing coefficients as soft targets [44], and joint frequency–temporal augmentation with reconstruction [45], and comparative work finds no consistent winner between contrastive and generative pretraining [46]. Pöppelbaum et al. apply SimCLR to fault classification on the Tennessee Eastman process and report gains at label fractions from 5% to 50% [47]. We adopt the standard objective with four domain-motivated augmentations as an ablation arm, and Section 4.4 reports it as a null result.

A separate strand asks not how to transfer but what to transfer from. Source-task selection treats the relevance of an auxiliary task to the target as a quantity to be estimated rather than assumed. Wang et al. weight auxiliary working conditions by their relevance to the target and sequence them from easier to harder during meta-training, on the principle that more relevant knowledge deserves more attention [48]. This literature bears directly on a bidirectional study, because it treats the source as a variable rather than a given and predicts that two directions between the same pair of plants need not behave alike. Section 5.2 examines whether the difference between our two directions originates in the source plant or in the target.

### 2.5. Positioning

The two programmes surveyed above leave a specific opening. Few-shot work demonstrates adaptation but rarely relocation; cross-dataset work demonstrates relocation but discards attack identity; and where both testbeds appear together, they are generally treated as two independent benchmarks rather than as source and target. The intersection, bidirectional transfer evaluated at the level of individual attack identifiers with no target-domain gradient updates, has not, to our knowledge, been reported for this plant pair.

A further gap emerges from our own results rather than from the literature. Because the inadequacy of a single centre is already understood, the prescription of additional centres has gone largely untested under distribution shift. Section 4.3 shows that it fails in the WADI-to-SWaT direction, and that a simpler local-neighbourhood rule succeeds there. The gap is not in the diagnosis but in the remedy. A third gap concerns attribution. Cross-plant results are rarely compared against conditions that carry no transferred information, so it is seldom clear whether a reported figure measures the representation or the task. Section 4.7 supplies such conditions for both recognition and detection.

## 3. Materials and Methods

### 3.1. Threat Model and Defender Resources

We consider an adversary who has compromised one or more programmable logic controllers, or the network paths linking them to field devices, and can spoof sensor readings, inject actuator commands that override supervisory logic, or coordinate manipulations across several process stages. These capabilities match the documented iTrust scenarios, which range from brief single-point spoofs such as SWaT Attack 14 on MV-303, lasting about five minutes, to sustained multi-stage manipulations such as SWaT Attack 28 on LIT-301 at roughly twenty-seven minutes. Several scenarios are built to resemble normal operation; WADI Attack 3 drains an elevated reservoir while spoofing the water-quality sensors that would expose it. The objective in each case is physical disruption, corruption of water quality, or persistence without detection.

Adaptive adversaries who alter behaviour in response to the detector fall outside this study, a constraint imposed by the data rather than chosen: the SWaT and WADI scenarios were executed against passive detection and contain no attacker-defender interaction loop. We also exclude denial-of-service against the PLC network or historian, attacks confined to the network layer without physical consequence (including the five scenarios in the SWaT list marked as having no physical impact, which carry no usable labels), and compromise of the historian software itself. The per-second stream is assumed authentic.

The resources available to the defender determine which results are operationally meaningful. A labelled attack corpus exists for a different facility, the source testbed. Unlabelled normal data from the target plant is available in quantity, since a running plant generates it continuously. Labelled target attacks are limited to a handful per class, five in our evaluation, of the kind obtainable from an incident write-up or red-team exercise, and a labelled target attack corpus cannot be produced, because attacks cannot be staged on live process equipment. We therefore evaluate recognition under a five-window budget and detection under reference sets spanning three orders of magnitude, so that each capability is measured against the resource it would draw on in deployment.

### 3.2. Data and Preprocessing

Both testbeds are operated by iTrust at the Singapore University of Technology and Design. SWaT [49] is a six-stage water-treatment process instrumented across 51 sensor and actuator channels; WADI [50] is a water-distribution testbed with 123. Each records per-second process state through a normal operating period and an attack period during which documented scenarios were executed against the physical plant.

**Figure 1:**
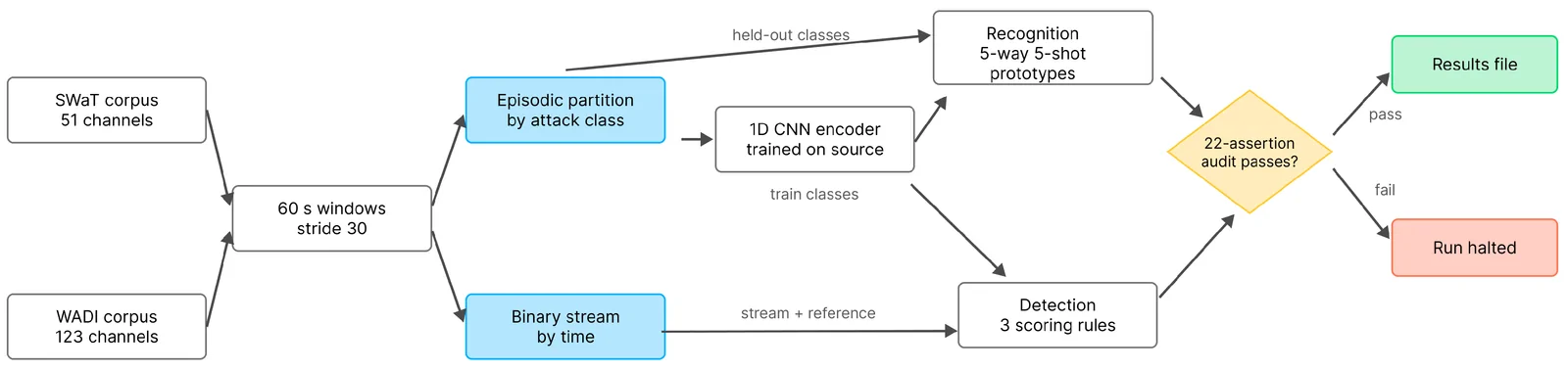
Study design. Both datasets are segmented into 60 s windows at stride 30 and partitioned twice: by attack class for the episodic recognition protocol and by time for the binary detection stream.

We segment both datasets into 60 s windows at stride 30, yielding 31,554 windows for SWaT (29,664 normal, 1890 attack, spanning 32 attack identities) and 31,906 for WADI (31,546 normal, 360 attack, 14 identities). Channels are normalised individually and clipped at ±20 standard deviations, bounding the influence of actuator transients without discarding them. For cross-plant evaluation, SWaT is zero-padded from 51 to 123 channels so that one encoder accepts input from either plant. Figure 1 illustrates the study design.

The stride imposes a constraint that shapes the evaluation design. Consecutive windows share half their raw timesteps, so any split assigning windows independently will place near-duplicates on both sides of its boundary, inflating measured performance in a way that leaves no trace in aggregate statistics. Both partitions described in Section 3.3 are constructed to respect this, and partition integrity is verified programmatically rather than assumed.

Attack identities are divided disjointly into episodic training, validation, and test sets, 16/6/9 for SWaT and 5/4/4 for WADI. The resulting test pools are small and heavily skewed (Table 1).

Attack 28 accounts for 89% of the SWaT test pool; WADI is less lopsided but works from a far smaller base. A 5-way 5-shot episode with *Q* = 10 queries requires 15 windows per class, and four classes fall below that threshold (SWaT 13, 29 and 31, and WADI 12). For every class, support windows are drawn first without replacement. Query windows are then drawn from the remaining windows of the class when at least *Q* remain; when fewer remain, as for these four classes, the sampler used for the reported results draws the query from the full class pool, so that some query windows can coincide with support windows. The expected share of query windows drawn from the support set is 83% for SWaT 29, 50% for SWaT 13, and 36% for SWaT 31 and WADI 12. Section 4.7 reports a re-evaluation under a strict sampler that draws the query only from the non-support remainder. WADI attack 12 is the consequential case, since an episode containing a normal class draws four attack classes and WADI’s pool contains exactly four, so that class appears in every episode of the SWaT-to-WADI direction.

This also makes the two transfer directions structurally unequal (Table 2). SWaT-to-WADI presents the same four-way discrimination in every episode, while WADI-to-SWaT draws a different four-class subset each time from 126 possibilities of varying difficulty. The directions therefore differ in average episode difficulty by construction, and their episode-to-episode variation measures different quantities, since variation in WADI-to-SWaT includes class-subset variation that is absent in the opposite direction. The two directions are reported separately throughout, and the difference between them is not treated as a measured effect.

### 3.3. Evaluation Protocol

Two complementary partitioning strategies were used to evaluate attack-type recognition and binary anomaly detection (Table 3). The episodic protocol partitions observations by attack identity and evaluates whether the encoder can discriminate previously unseen attack classes on a target plant. The binary-stream protocol partitions observations chronologically and evaluates whether normal and attack windows can be distinguished during a held-out operating period. These protocols address different transfer capabilities and therefore operate on different, although partially overlapping, subsets of each corpus.

Because neighbouring windows share half their observations, partition validity was established directly from the stored window indices. A programmatic verification protocol evaluated 22 conditions covering attack-identity disjointness across the episodic training, validation and test sets, temporal separation of the normal pools used for binary evaluation, and exclusion of self-supervised pretraining observations from all evaluation pools. An experiment proceeded only when every condition was satisfied.

Partition labels alone do not guarantee statistical separation between the underlying observations. In preliminary configurations, independently assigned overlapping windows left 91.2% of SWaT test windows and 91.4% of WADI test windows sharing half of their raw observations with a training neighbour, and the verification also detected reuse of a normal-reference pool across episodic samplers. Both conditions were removed before the reported experiments were run. Leakage control for overlapping time series must therefore be checked at the level of temporal indices rather than inferred from nominal partitions. The verification criteria, assertion outputs and partition indices are available from the authors on request.

### 3.4. Encoder, Scoring Rules, and Reproducibility

A 1D convolutional encoder maps each 60 × *C* window to a 128-dimensional embedding using grouped normalisation and concatenated mean-and-max pooling. Recognition follows the standard prototypical formulation: class prototypes are the means of the *K* support embeddings, and each query is assigned to its nearest prototype under Euclidean distance. We evaluate at 5-way 5-shot with *Q* = 10, with normal always among the five classes. For cross-plant evaluation the encoder is trained on source-plant episodes and applied to target-plant episodes with no gradient update on the target; the five labelled target windows per class serve only to form prototypes.

Binary detection reuses this encoder unchanged. A reference set of *R* normal windows is drawn from the target plant’s non-test period, and each window of the held-out stream receives an anomaly score under one of three rules, all computed over identical embeddings. The centroid rule scores by Euclidean distance to the mean of the reference embeddings, and is the convention inherited from the prototype and hypersphere literatures traced in Section 2.4. The local-density rule scores by mean distance to the *k* = 5 nearest reference embeddings. The nearest-mode rule scores by distance to the closest of *M* centres fitted by *k*-means with *M* selected by silhouette, and is included to distinguish two hypotheses that the literature tends to treat as one: that a single prototype is too few, and that any global prototype is the wrong object. Section 4.3 shows these have different answers. We fix *k* = 5 a priori and do not tune it, a choice examined by the sweep in Section 4.7.

The self-supervised arm pretrains the encoder with a SimCLR objective over normal windows drawn from the episodic-training partition only, using jitter, scaling, time-masking, and channel-masking augmentations, after which episodic training proceeds identically to the baseline arm. Training budget is matched across arms so that any difference is attributable to pretraining rather than compute.

Reproducibility is enforced by a gate requiring bitwise agreement across all recorded metrics between two executions of the same configuration in separate operating-system processes. Process separation is necessary rather than incidental: an equivalent check run twice within a single interpreter reports agreement while measuring only the reuse of cached kernel selections and allocator state. In our pipeline, twenty configurations passing that weaker check differed across sessions by up to 8.26 pp. We note this because in-process determinism checks are commonly written and return a confidently misleading result.

### 3.5. Experimental Design and Statistical Analysis

The seed is the unit of analysis and every comparison is paired on it. Compute is fixed at 10 epochs of 50 episodes, giving 500 episodic steps for every method and arm, with each run evaluated over 200 held-out test episodes. The primary arm runs at *n* = 39 seeds under enforced determinism; the self-supervised arm runs at *n* = 10 and is analysed separately rather than pooled.

The analysis plan was fixed before any result from the corrected pipeline existed, specifying the primary metric, unit of analysis, tests, multiplicity correction, equivalence margin, and the decision rules governing which claims each outcome would license. The plan and its deviation log are released with the paper. The log records that the study’s original headline hypothesis was withdrawn as untestable once the baseline it presupposed failed to replicate, and that two of four confirmatory family members were not implemented; the Holm correction is consequently applied over two *p*-values against a declared family of four, which is anti-conservative relative to the declaration.

Comparisons use paired two-sided *t*-tests at α = 0.05, with Cohen’s dz reported alongside. Multiplicity is controlled by Holm–Bonferroni across the confirmatory family. Equivalence claims are assessed by two one-sided tests at a ±2 pp margin fixed in advance; following standard TOST convention, this evaluates a 90% confidence interval against the margin, rather than the 95% interval used for point estimates elsewhere. The equivalence test was powered at *n* = 39 against an assumed true gap of zero, the best estimate available when the sample size was fixed; against the subsequently observed gap, it achieves a power of 0.458 rather than the intended 0.820, and we report this shortfall rather than extending the sample after the fact.

Confirmatory, descriptive, and exploratory results are labelled at the point of reporting. The mechanism analyses in Section 4.3 and Section 4.6 are exploratory, having been formulated after observing the detection result that prompted them. The reference conditions, neighbourhood-size sweep and actuator comparison in Section 4.7 were added during revision and are also exploratory. None of these analyses carries multiplicity control, and the intervals reported for them are unadjusted.

Five conventional binary baselines (random forest, gradient boosting, MLP, 1D-CNN, and a reconstruction autoencoder) are trained under a grouped, purged, stratified split: contiguous 50-window blocks assigned 70/15/15 and stratified on whether a block contains an attack, with boundary windows purged where adjacent overlapping windows would straddle a split. Purging removes windows from the training side only, leaving evaluation sets intact, and the resulting leak rate is zero by assertion. A purely chronological split is unavailable here, since WADI’s chronological training period contains no attack windows and no supervised classifier can be fitted on it. Cross-plant baselines are evaluated on the target’s test split so that both sides of each comparison share a denominator, at 5 seeds.

## 4. Results

### 4.1. Cross-Plant Attack-Type Recognition

Table 4 reports few-shot recognition performance across the four evaluation settings using 39 seeds under the process-level determinism procedure described in Section 3.4.

**Figure 2:**
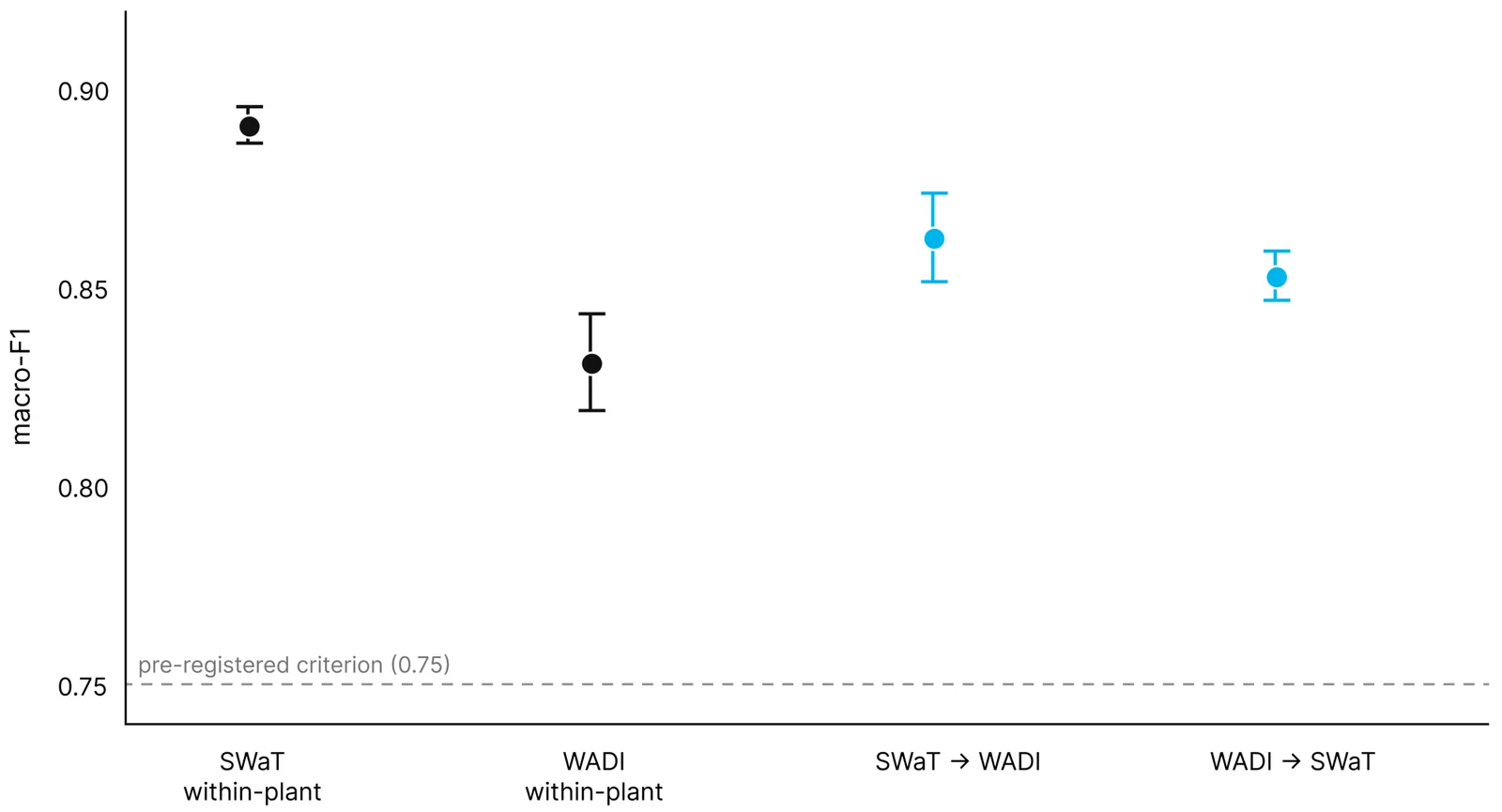
Attack-type recognition (5-way 5-shot macro-F1). Markers show mean and 95% CI over 39 seeds under enforced determinism; within-plant settings in black, cross-plant in blue. The dashed line marks the pre-registered criterion of 0.75.

Given five labelled windows per class and no gradient updates on the target plant, a source-trained encoder recognises held-out attack types at 0.8625 and 0.8528 macro-F1 (Figure 2). Holding the target plant fixed, transfer costs 3.80 pp on SWaT (0.8908 within-plant against 0.8528 transferred) and gains 3.15 pp on WADI (0.8310 against 0.8625). The pre-registered criterion for this claim, a 95% CI lower bound above 0.75 in at least one direction, is satisfied in both. Section 4.7 shows that these levels are also reached without transferred structure.

The gain on WADI does not indicate that transfer helps. WADI’s episodic training pool contains five attack classes and 107 attack windows, which leaves the within-plant condition data-starved, and Section 4.7 shows that an untrained encoder outperforms the WADI-trained one in this setting. The interpretable comparison holds the target plant fixed, and on SWaT transfer costs 3.80 pp.

Between directions, SWaT-to-WADI exceeds WADI-to-SWaT by 0.96 pp, 95% CI [−0.372, +2.299], *t*(38) = 1.461. The comparison is made only to test a 9.5 pp asymmetry reported in earlier work on this pair, which does not replicate under the corrected partitions; equivalence is not established either (Section 3.5), so we report non-replication and claim nothing further. The four cells reproduce to within −0.21, +2.36, +0.03 and −0.79 pp of earlier *n* = 10 estimates obtained on a different numerical backend.

### 4.2. Binary Detection Under the Centroid Rule

Binary detection was evaluated by constructing a prototype from 200 target-normal reference windows and using the distance from this prototype to score every window in the chronologically held-out target stream. Table 5 reports the resulting ROC-AUC values.

Centroid-based anomaly ranking was not preserved across plants. SWaT to WADI produced a ROC-AUC of 0.535, with an interval spanning 0.5, whereas WADI to SWaT produced 0.402, with the interval lying entirely below 0.5. On the full stream, attack windows in the second direction therefore tended to lie closer to the target-normal centroid than normal windows did. A ranking below chance indicates that the score still orders the two classes, but in the direction opposite to the one the detector assumes.

The size of this reversal depends on the composition of the attack stream. Because the episodic protocol partitions observations by attack identity and the binary protocol partitions them by time, the two draw on different attack populations. In the SWaT binary stream, 58% of attack windows carry the label −1, denoting mixed-attack or orphan windows that episodic recognition excludes, and only 10% belong to the held-out identities of the episodic test partition. Table 6 therefore restricts the attack stream progressively while leaving the normal test population unchanged.

Removing mixed and orphan windows moved the centroid ROC-AUC from 0.402 to 0.497, which removes the reversal, and restriction to held-out identities raised the point estimate to 0.571, although the interval remains wide because only 20 attack windows qualify. Stream composition therefore explains much of the sub-chance result, but it does not restore reliable detection under the centroid rule. The value of 0.402 is also specific to the encoder: applied to an untrained encoder, the same rule returns 0.528 (Table 7). A ranking below chance is therefore not treated here as a general property of cross-plant detection on this pair.

**Figure 3:**
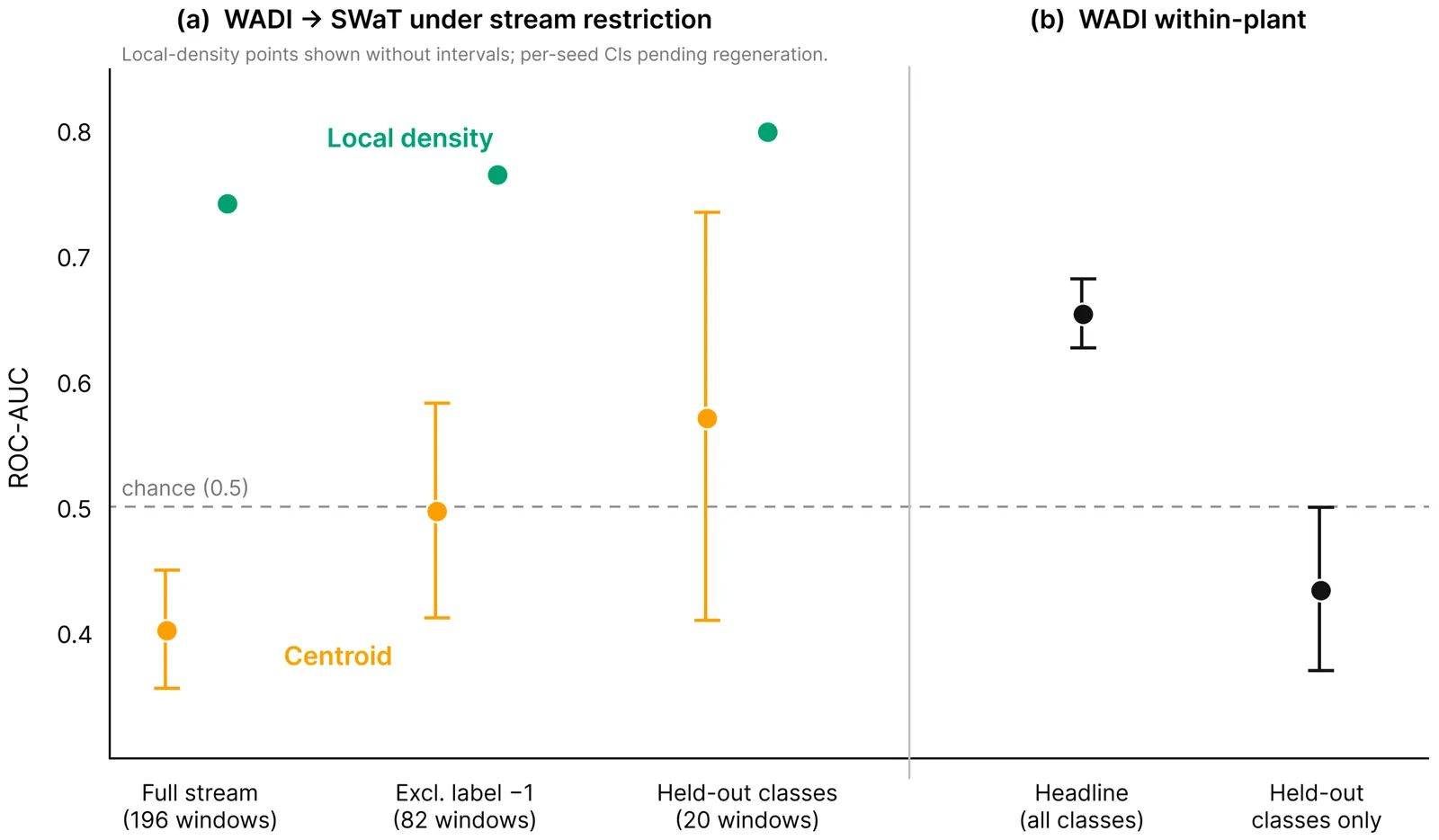
Stream composition determines what the headline number measures. (**a**) WADI → SWaT under progressive restriction of the evaluation stream: the centroid interval moves to span chance while local density improves. (**b**) WADI within-plant detection restricted to held-out attack classes falls below its headline value. Error bars are 95% CIs; local-density points are shown without intervals.

Local-neighbourhood scoring behaves differently (Figure 3). Its ROC-AUC rises from 0.742 on the full stream to 0.765 once mixed and orphan windows are removed and to 0.799 on held-out identities, so the same observations support much stronger ranking when normality is judged by proximity to observed target-normal embeddings rather than by distance from their mean.

The same target observations can therefore yield reversed, weak or useful rankings depending only on how the normal reference is summarised, and Table 7 shows that this holds for untrained as well as trained encoders. Section 4.3 examines this dependence directly.

### 4.3. Scoring-Rule Dependence of Cross-Plant Detection

Centroid failure can arise because the encoder no longer preserves information separating attack from normal behaviour or because that information is present but poorly expressed by distance from a single prototype. To distinguish the two, the scoring rule was varied while the encoder, embeddings, reference and evaluation stream were held constant (Table 8).

Replacing centroid distance with the mean distance to the five nearest target-normal embeddings increased the ROC-AUC by 0.340 (*d*_z_ = 3.09), and local-neighbourhood scoring outperformed the centroid rule in all ten seeds, with seed-level values from 0.603 to 0.844.

Because both rules operate on the same embeddings, the difference cannot be explained by representation learning, target-domain adaptation or a change in the evaluated observations. The embedding retains structure that is useful for anomaly ranking, but centroid distance does not express it. Section 4.7 shows that the same holds for an untrained encoder, so this structure is present in the target data rather than acquired from the source plant.

A nearest-mode rule tests whether the limitation arises from representing the normal class with a single centre. The target-normal reference contained, on average, 7.6 silhouette-selected clusters, yet scoring by distance to the nearest fitted centre produced a ROC-AUC of only 0.519 (95% CI [0.442, 0.596]). Adding global centres did not reproduce the performance obtained from the local relationships among reference embeddings, so the relevant distinction is not between one prototype and several but between summarising the reference by fitted mean locations and evaluating observations against its empirical local structure.

The influence of normal-reference size provides further evidence for this distinction. Table 9 reports performance in both directions as the number of target-normal reference windows increases from one to 500.

**Figure 4:**
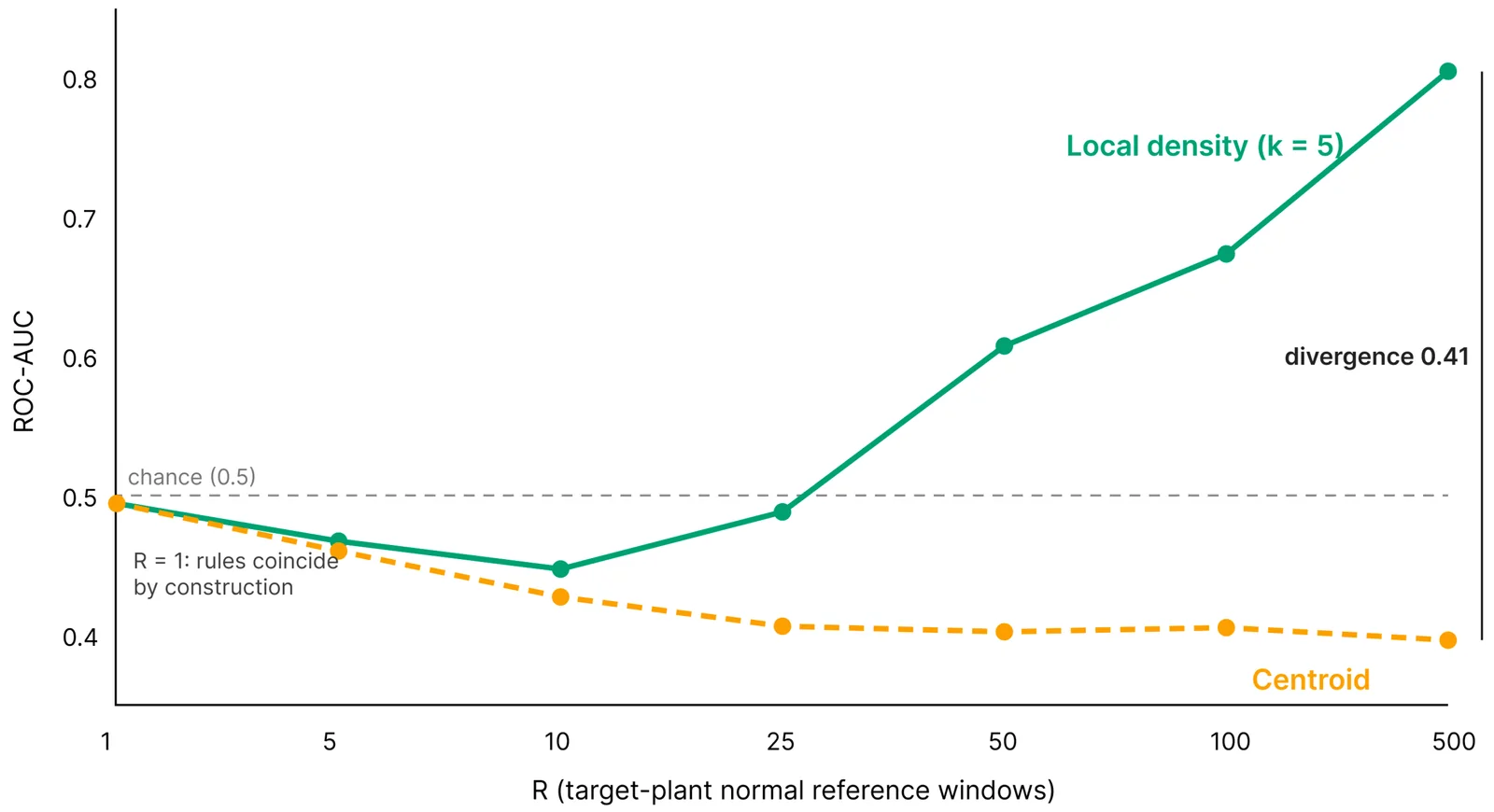
Detection ROC-AUC against normal-reference size *R*, WADI → SWaT. The two scoring rules coincide at R = 1 by construction and diverge as the reference grows: the centroid rule degrades while the local-density rule improves over identical embeddings, a divergence of 0.41 at R = 500.

At *R* = 1, the two rules reduce to the same distance calculation and produce the same ROC-AUC. In the WADI-to-SWaT direction, neither rule produces useful ranking up to *R* = 25. Beyond this point local-neighbourhood performance rises sharply, reaching 0.805 at *R* = 500, while centroid performance stays below chance and ends at 0.397 (Figure 4). Between *R* = 1 and *R* = 500, the centroid rule loses 0.098 (95% CI [−0.187, −0.008]) and the local rule gains 0.311 (95% CI [+0.256, +0.365]). Additional normal observations improve detection only when the scoring rule preserves the information they contribute.

The two trajectories reflect a difference in what the rules estimate. The centroid uses additional observations to refine a global mean; the local rule uses them to cover more of the regions in which normal observations occur. For a compact, unimodal normal class, a better estimate of the mean should give a more stable reference, and the decline observed here indicates that this assumption does not hold. Section 5.2 develops this account.

The opposite direction produces no separation. For SWaT to WADI, centroid scoring reaches 0.534 (95% CI [0.473, 0.595]) and local-neighbourhood scoring reaches 0.513 ([0.446, 0.580]), a difference of −0.021 whose interval spans zero. Across reference sizes, the two rules stay between 0.504 and 0.536 (Table 9), so neither rises clearly above chance. The scoring-rule effect is therefore not general to this pair of plants, and Table 7 indicates that it follows the target plant rather than the transfer direction: the separation is large whenever SWaT is the target and the encoder was not trained on SWaT, small for the SWaT-trained encoder, and small or absent whenever WADI is the target.

### 4.4. Self-Supervised Pretraining

Table 10 reports the pretraining ablation across all four settings.

The estimated improvement ranged from 0.37 to 2.37 percentage points. The largest transfer effect occurred for WADI to SWaT, where pretraining increased macro-F1 by 1.39 percentage points (*d*_z_ = 0.70); this approached the unadjusted threshold (*p* = 0.055) but did not remain significant after Holm adjustment. The SWaT-to-WADI effect was smaller, at 0.37 percentage points.

All four estimates are positive, which suggests that pretraining on unlabelled normal dynamics may give a modest initialisation advantage, but the largest estimate occurred within WADI rather than across plants, so the effect is not specific to cross-plant adaptation. Because Section 4.7 shows that recognition at these levels does not require transferred structure, a small increment from pretraining is also expected to be difficult to detect on this protocol. The results support neither a reliable improvement nor equivalence between the pretrained and non-pretrained conditions.

### 4.5. Conventional Supervised Baselines

Table 11 reports attack-class F1 for five conventional binary detection models under the purged split described in Section 3.5.

Within-plant performance was higher than cross-plant performance but remained moderate: the best attack-F1 was 0.729 on SWaT, from random forest and the autoencoder, and 0.464 on WADI, from random forest. Temporal separation and attack imbalance make binary detection harder than randomly mixed window partitions suggest. Cross-plant transfer produced a sharper decline. From SWaT to WADI, no baseline exceeded an attack-F1 of 0.082, and the uniformly low values across tree-based, neural and reconstruction-based models show that the failure is not specific to one model family.

Transfer from WADI to SWaT was less uniform. Random forest, gradient boosting and the multilayer perceptron reached at most 0.091, whereas the autoencoder reached 0.416. The convolutional network’s mean of 0.341 conceals marked partition sensitivity: its five values were 0.741, 0.704, 0.047, 0.059 and 0.152, two successful outcomes and three failures depending on which contiguous attack segments entered each partition.

This sensitivity reflects a property of the datasets. Attacks occur in long contiguous episodes, with SWaT attack 28 alone contributing 1140 consecutive windows, so assigning blocks to partitions changes the attack identities and operating conditions in each split as well as their number. Within-plant attack-F1 consequently varied by as much as 0.44 across valid splits, and because the random forest used a fixed internal seed, that variation arose from partition composition rather than initialisation. A single split and point estimate cannot adequately characterise binary detector performance on these datasets.

### 4.6. Within-Plant Detection and Class Composition

Attack-class composition also influenced detection performance within a single plant. WADI within-plant detection achieved a headline ROC-AUC of 0.654 when evaluated on the complete chronological stream. When the same analysis was restricted to attack identities held out from episodic training, ROC-AUC decreased to 0.434, with a 95% confidence interval of [0.369, 0.499].

The full-stream result was therefore carried largely by attack identities represented during encoder training, an advantage no cross-plant setting can have. Comparing within-plant and cross-plant scores without aligning attack-class composition attributes both representation shift and class familiarity to plant transfer. Once evaluation is restricted to held-out identities, the within-plant advantage largely disappears, and on WADI the centroid rule falls below chance, so its limitation appears within a single plant as well as across plants.

### 4.7. Comparison Against Untrained and Feature-Space References

The recognition results in Section 4.1 show that a source-trained encoder supports attack-type discrimination on an unseen plant but not how much of that performance depends on the transfer. Three reference conditions were therefore evaluated on exactly the same episodes, support sets and query sets. The first replaces the trained encoder with an untrained network of identical architecture and random initialisation. The second removes the encoder, classifying standardised flattened windows by nearest class mean, with standardisation fitted on the support set so that the comparison remains inductive. The third keeps the trained encoder but randomly permutes the channel order of the target plant before inference, removing any correspondence between channel positions in the two testbeds. These comparisons were run on the saved encoders at *n* = 10 seeds and 200 test episodes as a separate paired evaluation with its own trained column. Values in that column therefore differ from the 39-seed estimates in Table 4, and all comparisons in this section are made within Table 12 and Table 13.

In neither cross-plant direction does the trained encoder differ detectably from an untrained network of the same architecture: the differences are −0.009 and +0.006, with intervals spanning zero, and the trained encoder is the better of the two in four and seven of ten seeds, respectively. The intervals rule out an advantage of source training larger than 0.018 from SWaT to WADI and 0.020 from WADI to SWaT, so any contribution of source training to cross-plant recognition is small. Source training is clearly beneficial only within SWaT, where it adds 0.053 macro-F1 in every seed; within WADI, the trained encoder is the weaker of the two.

Permuting the channel order of the target plant leads to a similar conclusion (Table 12). The permutation changes macro-F1 by −0.041 (95% CI [−0.098, +0.017]) from SWaT to WADI and by −0.042 ([−0.070, −0.014]) from WADI to SWaT, so about ninety-five per cent of cross-plant performance survives the loss of positional correspondence. Within SWaT, on native unpadded channels, it costs 0.131 ([−0.177, −0.084]), whereas within WADI it changes macro-F1 by −0.007 with an interval spanning zero, so channel identity carries information in one native setting but not measurably in the other. The padded input explains why little of the cross-plant result depends on it: channels 51 to 122 of a SWaT window are identically zero by construction, while slightly more than half of WADI’s signal mass lies in that range, so from SWaT to WADI most of the target signal passes through weights that encountered only zeros during training.

The five-way episodic protocol on these corpora is therefore solvable to about 0.86 macro-F1 without transferred structure, and the recognition figures in Table 4 measure the difficulty of that protocol rather than the quality of the transferred representation. This does not invalidate the protocol, which remains a reasonable way to pose the attack-identity question, but it bounds what performance on it can demonstrate. The sparse-class sampling described in Section 3.2 has little effect on these conclusions. Re-evaluation with the strict sampler changed macro-F1 by −0.0004 within SWaT, −0.0002 within WADI and −0.0022 from WADI to SWaT, with all intervals spanning zero. From SWaT to WADI, the strict and original samplers returned identical values in every seed, so that setting is not counted as verified.

Detection behaves differently from recognition under the same reference conditions. The separation between the two scoring rules in the WADI-to-SWaT direction is present with an untrained encoder, at +0.269 (95% CI [+0.191, +0.347]), and is therefore not a property of the transferred representation. Source training affects the two rules unequally: within SWaT, it improves centroid scoring by 0.185 while leaving local-neighbourhood scoring unchanged (−0.037, with an interval spanning zero). Training shapes the global geometry of the embedding, on which the centroid depends, and has comparatively little effect on the local neighbourhood structure on which the better rule depends.

The local-neighbourhood rule was applied with *k* = 5 throughout, a value fixed before any detection result was examined. Table 14 reports the full sweep over *k* against the same 200-window reference, with the centroid rule as a fixed comparator.

The separation from centroid scoring in the WADI-to-SWaT direction exceeds 0.2 ROC-AUC for every *k* between 1 and 20 and remains distinguishable from zero at *k* = 50, so the effect is not specific to the value chosen; these intervals are unadjusted across the sweep. The sweep also shows that *k* = 5 was a conservative choice, since *k* = 1 and *k* = 2 both reach 0.767. It is retained in the preceding tables because changing it after inspecting the sweep would convert an untuned parameter into a tuned one. The decay of the effect as *k* grows is informative. As *k* approaches the size of the reference, the score becomes an average over the whole reference and the advantage disappears: at *k* = 200, the rule returns 0.408 against the centroid’s 0.402. What separates the two rules is locality, and removing the locality removes the difference. The two settings that show no separation at *k* = 5 show none at any *k*.

A further question is whether the modes on which the local rule depends correspond to anything physical. SWaT records 26 discrete actuator channels, namely motorised valves, pumps and the UV dechlorinator, whose configuration over a window describes what the plant is doing. For each seed, the 200-window target-normal reference was embedded with the WADI-trained encoder, clustered at the silhouette-selected number of clusters, and compared against the actuator configuration recovered from the raw record. Table 15 summarises the agreement.

Agreement is above chance but partial, so the clusters are not a relabelling of actuator states. In one seed, shown for illustration, seven of the 26 actuators differ in modal state between clusters, and three of the nine clusters correspond to distinct actuator configurations, the clearest being one in which MV101, MV201, P101, P203 and P205 all sit opposite to the majority, corresponding to the first two treatment stages being largely shut down. The remaining six clusters share the dominant configuration and are separated by continuous process state: their mean levels in the three tanks span 100.3, 103.4 and 122.7 raw units against corpus standard deviations of 125.0, 92.6 and 147.9, placing them at different phases of the fill and drain cycle. The modes therefore correspond to operating states in two senses, which equipment is running and where the process sits in its cycle. This comparison was made for the SWaT target, where the local rule succeeds.

## 5. Discussion

### 5.1. Attributing Cross-Plant Performance

Recognition and detection were evaluated under different supervision, class populations and task designs, so a direct ranking between them is not something this study can support. Recognition uses five labelled windows from each target class and detection a normal reference with no attack labels; the protocols partition the corpora by attack identity and by time, respectively, so that on SWaT only one of nine attack identities is common to both evaluation sets, and the restriction analysis in Section 4.6 addresses only the difference in class population. The comparison is therefore stated in terms of what each capability requires from the target plant. Within that framing, Section 4.7 shows that the attack identities in these corpora are separable in a five-way episode by geometry that does not require learning on a source plant.

Recognition is a relative decision. Given a few labelled target examples, a model needs only to decide which supported class a window most resembles, and on this protocol, even a random projection preserves enough of that ordering. Binary detection asks a different question, whether an observation belongs to the target plant’s normal regime at all, which requires an estimate of that regime’s scale, location and operating states. A small labelled attack set cannot supply that estimate, because the information detection needs is not contained in attack-class separation.

This has a direct consequence for how cross-dataset detection results are read. Poor transfer of a binary detector does not by itself show that the representation has lost discriminative information, because degradation of the representation and degradation of the decision rule are separate failure modes. In the present data, the decision rule dominated: over trained and untrained embeddings alike, the choice of rule changed WADI-to-SWaT ROC-AUC by more than 0.26. Much of the cross-dataset literature reviewed in Section 2.2 evaluates transfer only through binary detection and would not distinguish these cases.

### 5.2. Representing Normal Operation Across Plant States

The centroid rule assumes that normal behaviour can be represented by one central point. The assumption is reasonable when observations gather around a single operating condition, but industrial plants move through several legitimate states, such as filling, draining, backwashing and standby, and each produces its own pattern of sensor readings and actuator positions.

In the embedding space these states occupy separate regions, and their average can fall between them, in a place that corresponds to no state in which the plant operates. A window can then be far from the average while close to a valid state, and an attack that moves some measurements towards the centre of the combined states can lie near the average. Adding normal windows improves the estimate of the average without making it a better description of the plant, which is why the centroid becomes more precisely located while its value for detection declines (Table 9). Local-neighbourhood scoring uses the same windows differently: it asks whether a new observation is close to any example of normal behaviour, and as the reference grows it covers more of the plant’s operating states.

The nearest-mode result qualifies the standard remedy. Previous work addresses the weakness of a single centre by fitting several centres [37,38,39,40], but that approach did not recover detection here. Each fitted centre still replaces a group of observations by an average point, which is inadequate when operating states differ in shape and density, overlap, or are connected by gradual transitions. The claim supported by these results is correspondingly narrow: summarising the reference by one or more mean locations under Euclidean distance fails on this target, while keeping the local structure of the reference succeeds. The results do not show that every parametric description of normal operation fails.

The same comparison explains why prototypes can serve recognition while failing detection. In recognition, several class prototypes are compared and the decision is relative; in detection, a single normal prototype must define normality by itself, and its distance must carry the same meaning across operating states and after transfer. Success in few-shot classification does not guarantee that requirement.

Normal operation should therefore be represented through the states the plant actually occupies. Local-neighbourhood distance is one way to do this; models of operating states, local density, temporal transitions or the shape of the normal region may do it better. The representation of normality is a design problem in its own right, separate from the encoder.

Two further results support this account. The separation between the rules persists under an untrained encoder at more than 0.26 ROC-AUC, so it does not depend on the transferred representation; and the advantage of the local rule is an advantage of locality, since it disappears as the neighbourhood grows, returning 0.408 at *k* = 200 against the centroid’s 0.402 (Table 14). The modes also correspond to identifiable plant behaviour. Embedding clusters agree with the configuration of the 26 discrete actuator channels above chance (adjusted mutual information 0.283, Table 15); some correspond to distinct equipment configurations, such as the first two treatment stages being shut down, while others share a configuration and differ in tank level by about one corpus standard deviation, at different phases of the fill and drain cycle. The mean of such a distribution averages tank levels across filling and draining under an averaged equipment configuration, a configuration unlikely to occur in normal operation, which is why distance from it says little about whether a window is anomalous.

The account applies to the direction in which detection is recoverable. From SWaT to WADI, no rule we tested rises clearly above chance with either trained or untrained embeddings, and within WADI, the two rules perform alike, so the explanation above does not account for detection on WADI. The pattern in Table 7 is also informative about its cause. The source-task relevance literature [48] would lead one to expect differences between directions to originate in the source plant. Here, the separation appears whenever SWaT is the target, including with untrained encoders, and it is small or absent whenever WADI is the target, which points to properties of the target plant’s normal operation rather than to the relevance of the source. Whether WADI’s normal operation lacks the separable modes found in SWaT, or whether its attacks lie within those modes, is not resolved by these experiments.

### 5.3. Implications for Cross-Plant Detection and Evaluation

A cross-plant detector must separate what can be learned from other facilities from what must be estimated at the target plant. Normal behaviour, score interpretation and alarm thresholds remain tied to the environment in which they are measured, and in the present evidence, even the representation contributes little that a randomly initialised encoder does not.

The autoencoder baseline shows the same problem at the thresholding stage. Its WADI-to-SWaT attack-F1 of 0.416 exceeded those of the discriminative baselines, so reconstruction error retained some information after transfer, yet a threshold set at the 99th percentile of source reconstruction errors has no fixed meaning once sensor scales, dynamics and operating states change. Centroid scoring and source-based thresholding fail at different points but rest on the same assumption, that a quantity learned from one distribution keeps its meaning in another.

Target-normal data offer a practical way out. A running plant produces normal observations continuously even when labelled attacks are unavailable, and these can describe its operating states, set the scale of anomaly scores and fix thresholds for the target environment. Cross-plant deployment need not avoid target adaptation; it needs to avoid adaptation that depends on large collections of labelled target attacks. Attack recognition can then be anchored by a small labelled support set, normal behaviour described from unlabelled target observations, and calibration carried out within the target plant, each estimated from the data that contain the information it requires.

Evaluation should preserve the same distinctions. ROC-AUC shows whether a score ranks attacks above normal observations, but not whether a chosen threshold yields an acceptable alarm rate, and attack-F1 changes sharply with prevalence and attack composition. Ranking, thresholded detection, false-alarm burden and event-level performance should be reported separately, consistent with evidence that detector rankings on SWaT and WADI change under an alarm budget [32].

Evaluation outcomes on these datasets also depend on how attack episodes are divided. Because attacks occur in long contiguous periods, a split determines which attacks and operating conditions appear in training and testing, which is why attack-F1 varied by as much as 0.44 across leakage-free splits. Results should be reported across several valid temporal partitions with the attack identities in each, and within-plant and cross-plant results should be compared for matched attack populations. They should also be reported against reference conditions of the kind used in Section 4.7, since without them a recognition figure of 0.86 on this protocol would be read as evidence of transfer.

### 5.4. Limitations

The findings should be read within the boundaries of the data. SWaT and WADI are both water systems built within the same testbed programme at one institution, so every conclusion is a single observation at the plant-pair level, and the two directions of that single pair already behave differently. Transfer between them is more demanding than evaluation within one dataset, but it does not represent the variation found across power, manufacturing, oil and gas, or operational water plants. Some findings are expected to travel and some are not. The degradation of a centroid summary as the reference grows is a property of the scoring rule and of multimodal geometry, and it should appear wherever normal operation is multimodal, although it has been shown here for one target plant only. The reported magnitudes, including the recognition levels and the size of the scoring-rule separation, are properties of these two plants and should not be treated as transferable quantities.

Attack availability limits the recognition estimates. Several classes contain fewer windows than an episode requires, so their query windows repeat within and across episodes, and a large number of episodes does not yield an equally large number of independent attack observations. The restricted detection analysis rests on only 20 SWaT attack windows from held-out identities. Intervals across seeds capture variation in training and episode sampling, not independent attack events collected under different conditions.

The five-shot setting assumes that five labelled target windows exist for every attack class in an episode. This is realistic when an attack has already been identified through an incident investigation, laboratory exercise or trusted intelligence source, but not for an attack whose identity is still unknown. The results support rapid classification from a small labelled reference set, not identification of an attack for which no labelled target example exists.

Padding SWaT from 51 to 123 channels lets one encoder process both datasets but does not make their channels equivalent. The permutation test in Section 4.7 shows that little of the cross-plant result depends on positional correspondence; shared-variable experiments and channel-independent encoders remain untested.

The scoring-rule effect was observed for one target plant. The separation is present whenever SWaT is the target and small or absent whenever WADI is the target, so it cannot yet be treated as a general property of industrial transfer. The sweep over *k* shows that the effect does not depend on the neighbourhood size chosen, but only three scoring rules were compared, and local outlier factors, kernel density estimates, state-conditioned models and temporal scoring methods would give a stronger account of which properties produce the improvement.

The explanation based on multiple operating states is supported by the cluster structure, by the opposite responses of the two rules to additional normal data, by the decay in the local advantage as *k* grows, and by the partial agreement between clusters and actuator configurations. That agreement is moderate (adjusted Rand index 0.111), the actuator comparison was made for the SWaT target only, and the study does not measure whether the centroid lies in a region rarely occupied by normal windows. A direct measurement of density around the centroid would test the explanation more closely.

Binary detection was assessed through window-level metrics, which do not show how scores combine into alarms, how quickly an attack is detected, or how many alerts an operator must investigate. Detection delay, alarm duration, false alarms per hour and performance under a fixed alarm budget are needed before operational suitability can be judged.

Finally, additional seeds improve estimates of computational variation but do not add plants, attack campaigns or independently observed operating environments. Broader claims require replication on independently collected industrial data, with balanced transfer tasks and larger rare-attack samples.

## 6. Conclusions

This study examined whether knowledge learned from one industrial control testbed can support attack recognition and binary detection on another, under partitions whose integrity was verified programmatically. The results locate the transferable component more narrowly than the framing of previous cross-dataset work suggests.

With five labelled target windows per class and no target-domain gradient updates, the source-trained encoders achieved macro-F1 scores of 0.8625 for SWaT to WADI and 0.8528 for WADI to SWaT. An untrained encoder of the same architecture reached the same level, a classifier with no encoder came close to it, and permuting the target channel order changed it little. These values therefore characterise the episodic protocol on these corpora rather than the quality of the transferred representation.

Detection was governed by the choice of scoring rule and by the target plant. When SWaT was the target, a local-neighbourhood rule exceeded centroid scoring by 0.340 ROC-AUC over identical embeddings and by 0.269 over untrained ones; from SWaT to WADI, neither rule rose clearly above chance at any reference size. The centroid value of 0.402 is specific to this encoder and evaluation stream, moving to 0.497 when mixed and orphan windows are excluded and to 0.528 under an untrained encoder, and a ranking below chance is not presented here as a general property of cross-plant detection.

The normal-reference experiment showed why the choice of rule matters. At *R* = 500, the local rule reached 0.805 while the centroid fell to 0.397: additional normal data helped when they increased coverage of the plant’s operating states and did not help when reduced to a global mean.

These findings support separating three parts of cross-plant detection: learning a representation, describing normal operation in the target plant, and converting scores into decisions. Based on this evidence, the second and third parts, which draw on unlabelled normal data and calibration within the target plant, carry most of the weight, and the first contributes less than its prominence in the literature suggests.

The evidence is limited to one pair of water testbeds, and the scoring-rule effect to one target plant. The geometric explanation should be tested on additional plants, industrial sectors and scoring rules, including a direct measurement of density around the centroid, and detection should be evaluated through complete alarm behaviour rather than window-level ranking alone.

Within these limits, the study supports a clear conclusion. Cross-plant results on these benchmarks are sensitive to design choices ordinarily treated as incidental, including the scoring rule, the size of the normal reference, and the presence of a representation-free baseline, and they are best reported alongside the reference conditions that establish what they measure.

## Figures and Tables

**Table 1:** Test-pool composition by attack identity.

SWaT ID	2	8	13	14	23	28	29	31	36	Total
Windows	17	34	10	16	25	1140	6	14	20	1282
**WADI ID**	**2**		**3**		**7**		**12**		**Total**	
Windows	22		55		25		14		116	

**Table 2:** Structural asymmetry between transfer directions.

Direction	Test Attack Classes	Classes per Episode	Class Sampling
SWaT → WADI	4	all 4	none; a fixed problem
WADI → SWaT	9	4 of 9	126 possible subsets

**Table 3:** The two partitions and their scope.

Partition	Partitioned b4.	Governs
Episodic protocol	Attack class (disjoint train/val/test identities)	Recognition, Section 4.1
Binary stream	Time (win_train/win_val/win_test)	Detection, Section 4.2 and Section 4.3

**Table 4:** Attack-type recognition, 5-way 5-shot macro-F1 (*n* = 39, enforced determinism).

Setting	Mean Macro-F1	SD	95% CI
SWaT within-plant	0.8908	0.0142	[0.8862, 0.8954]
WADI within-plant	0.8310	0.0376	[0.8188, 0.8432]
SWaT → WADI	0.8625	0.0344	[0.8513, 0.8736]
WADI → SWaT	0.8528	0.0191	[0.8466, 0.8590]

**Table 5:** Binary detection ROC-AUC under the centroid rule (*n* = 10).

Encoder → Stream	ROC-AUC	95% CI
SWaT within-plant	0.724	[0.694, 0.753]
WADI within-plant	0.654	[0.626, 0.681]
SWaT → WADI	0.535	[0.475, 0.596]
WADI → SWaT	0.402	[0.355, 0.449]

**Table 6:** WADI → SWaT detection under progressive stream restriction, centroid rule.

Stream	Attack Windows	ROC-AUC	95% CI
Full	196	0.402	[0.355, 0.449]
Excluding label −1	82	0.497	[0.411, 0.582]
Held-out classes only	20	0.571	[0.409, 0.734]

**Table 7:** Detection ROC-AUC under both scoring rules, trained and untrained encoders (*n* = 10, 200-window reference). Values come from a separate evaluation of the saved encoders and differ from Table 5 by at most 0.002.

Setting	Encoder	Centroid	Local Neighbourhood	Difference
SWaT within-plant	trained	0.723	0.782	+0.059
SWaT within-plant	untrained	0.539	0.819	+0.280
WADI within-plant	trained	0.656	0.652	−0.003
WADI within-plant	untrained	0.517	0.541	+0.024
SWaT → WADI	trained	0.534	0.513	−0.021
SWaT → WADI	untrained	0.480	0.543	+0.063
WADI → SWaT	trained	0.402	0.742	+0.340
WADI → SWaT	untrained	0.528	0.797	+0.269

**Table 8:** Scoring-rule comparison over identical embeddings, WADI → SWaT (*n* = 10).

Metric	Centroid	Local Density (*k* = 5)	Δ	95% CI
ROC-AUC	0.402	0.742	+0.340	[+0.262, +0.419]

**Table 9:** Detection ROC-AUC against normal-reference size *R*, both directions (*n* = 10).

*R*	1	5	10	25	50	100	500
WADI → SWaT, local density	0.495	0.468	0.448	0.489	0.608	0.674	0.805
WADI → SWaT, centroid	0.495	0.461	0.428	0.407	0.403	0.406	0.397
SWaT → WADI, local density	0.511	0.504	0.509	0.506	0.536	0.514	0.507
SWaT → WADI, centroid	0.511	0.507	0.520	0.523	0.530	0.530	0.533

**Table 10:** Self-supervised pretraining effect on recognition macro-F1 (*n* = 10, Holm–Bonferroni).

Setting	Δ (SSL − no-SSL)	95% CI	*p*	Holm-Adjusted
WADI → SWaT (H2a)	+1.39 pp	[−0.04, +2.82]	0.055	0.111
SWaT → WADI (H2b)	+0.37 pp	[−2.86, +3.60]	0.799	0.799
SWaT within-plant (exploratory)	+1.39 pp	[−0.62, +3.40]	0.153	-
WADI within-plant (exploratory)	+2.37 pp	[−2.36, +7.10]	0.286	-

**Table 11:** Supervised baseline attack-F1 under the leak-free grouped split (*n* = 5).

Setting	RF	XGB	MLP	CNN	AE
SWaT within-plant	0.729	0.610	0.640	0.638	0.729
WADI within-plant	0.464	0.293	0.389	0.430	0.349
SWaT → WADI	0.022	0.014	0.016	0.082	0.038
WADI → SWaT	0.091	0.000	0.042	0.341	0.416

**Table 12:** Recognition macro-F1 against reference conditions (*n* = 10 seeds, 200 episodes; separate paired evaluation, see text).

Setting	Trained Encoder	Untrained Encoder	Feature Space, No Encoder	Permuted Target Channels
SWaT within-plant	0.9122	0.8593	0.8624	0.7813
WADI within-plant	0.8298	0.8784	0.8206	0.8228
SWaT → WADI	0.8615	0.8705	0.8206	0.8210
WADI → SWaT	0.8705	0.8642	0.8625	0.8284

**Table 13:** Paired differences between the trained encoder and each reference condition.

Setting	Trained − Untrained	95% CI	Trained − Feature Space	95% CI
SWaT within-plant	+0.0528	[+0.0372, +0.0684]	+0.0497	[+0.0356, +0.0639]
WADI within-plant	−0.0487	[−0.0823, −0.0150]	+0.0092	[−0.0178, +0.0362]
SWaT → WADI	−0.0090	[−0.0361, +0.0182]	+0.0409	[+0.0210, +0.0608]
WADI → SWaT	+0.0063	[−0.0073, +0.0198]	+0.0081	[−0.0036, +0.0198]

**Table 14:** Detection ROC-AUC against the neighbourhood size *k* (*n* = 10, 200-window reference). Centroid values come from the same separate evaluation as Table 7.

Setting	Centroid	k = 1	k = 2	k = 5	k = 10	k = 20	k = 50	k = 100	k = 200
SWaT within-plant	0.723	0.768	0.779	0.782	0.778	0.768	0.748	0.732	0.725
WADI within-plant	0.656	0.645	0.648	0.652	0.654	0.657	0.659	0.659	0.656
SWaT → WADI	0.534	0.504	0.508	0.513	0.517	0.518	0.521	0.522	0.529
WADI → SWaT	0.402	0.767	0.767	0.742	0.692	0.613	0.480	0.425	0.408

**Table 15:** Agreement between embedding clusters and actuator configurations, WADI → SWaT (*n* = 10 seeds).

Quantity	Mean	95% CI
Clusters selected, k	7.6	[5.28, 9.92]
Distinct actuator configurations	20.7	[18.3, 23.1]
Adjusted mutual information	0.283	[0.212, 0.354]
Adjusted Rand index	0.111	[0.038, 0.183]
Cluster purity	0.649	[0.618, 0.680]

## Data Availability

Both datasets are third-party and available from iTrust, Singapore University of Technology and Design, under their data request process; we redistribute no raw data. All analysis code, the partition audit, the determinism gate, the pre-registration and its deviation log, per-seed results, and the figure-generation scripts are available from the corresponding author on request.
